# Macular retinal and choroidal thickness in unilateral amblyopia using swept-source optical coherence tomography

**DOI:** 10.1186/s12886-017-0559-3

**Published:** 2017-09-15

**Authors:** Syunsuke Araki, Atsushi Miki, Katsutoshi Goto, Tsutomu Yamashita, Go Takizawa, Kazuko Haruishi, Yoshiaki Ieki, Junichi Kiryu, Kiyoshi Yaoeda

**Affiliations:** 1 Department of Ophthalmology, Kawasaki Medical School, 577 Matsushima, Kurashiki, Okayama, 701-0192 Japan; 2 Department of Sensory Science, Faculty of Health Science and Technology, Kawasaki University of Medical Welfare, 288 Matsushima, Kurashiki, Okayama, 701-0193 Japan; 3 Yaoeda Eye Clinic, 2-1649-1 Naga-Chou, Nagaoka, Niigata, 940-0053 Japan

**Keywords:** Amblyopia, Retinal thickness, Choroidal thickness, Optical coherence tomography

## Abstract

**Background:**

To investigate macular retinal and choroidal thickness in amblyopic eyes compared to that in fellow and normal eyes using swept-source optical coherence tomography (SS-OCT).

**Methods:**

This study examined 31 patients with hyperopic anisometropic amblyopia (6.9 ± 3.8 years, mean ± standard deviation), 15 patients with strabismic amblyopia without anisometropia (7.9 ± 4.2 years), and 24 age-matched controls (7.8 ± 3.3 years). Retinal and choroidal thickness was measured by 3D scans using SS-OCT. A 6-mm area around the fovea was automatically analyzed using the Early Treatment Diabetic Retinopathy Study map. The thickness from SS-OCT was corrected for magnification error using individual axial length, spherical refraction, cylinder refraction, and corneal radius. Retinal thickness was divided into the macular retinal nerve fiber layer (mRNFL), ganglion cell layer + inner plexiform layer (GCL+IPL), ganglion cell complex (GCC), and the inner limiting membrane to the retinal pigment epithelium (ILM-RPE) thickness. Retinal and choroidal thickness was compared among amblyopic, fellow, and normal eyes.

**Results:**

In both amblyopia groups, there was no significant difference in the mRNFL, GCL+IPL, and GCC thicknesses among the amblyopic, fellow, and control eyes. In the anisometropic amblyopia group, choroidal thickness (subfovea, center 1 mm, nasal and inferior of the inner ring, nasal of the outer ring, and center 6 mm) of amblyopic eyes were significantly greater than that of fellow and normal eyes. In contrast, none of the choroidal thicknesses were significantly different among the investigated eyes in the strabismic amblyopia group.

**Conclusions:**

We found no significant difference in inner retinal thickness in patients with unilateral amblyopia. Although there were significant differences in choroidal thickness with hyperopic anisometropic amblyopia, there was no significant difference for the strabismic amblyopia. The discrepancy in choroidal thickness between the two types of amblyopia may be due to both differences in ocular size and underlying mechanism.

## Background

Amblyopia, which is a visual disorder characterized by subnormal visual acuity (VA) and contrast sensitivity in one or both eyes, is caused by either visual deprivation or abnormal binocular interactions [1]. Many studies that investigated the pathogenesis of amblyopia, including animal experiments by Hubel and Wiesel in the 1960s, have found morphological and functional abnormalities in the visual cortex and lateral geniculate nucleus [2–4]. In recent years, dysfunction in the lateral geniculate nucleus as well as in the visual cortex has also been found in human amblyopes [5, 6].

On the other hand, Ikeda [7] performed neurophysiological experiments in cats and reported that underdevelopment of the retinal ganglion cells was associated with amblyopia. Furthermore, an electrophysiological experiment reported finding a functional disturbance of the retina in human amblyopes [8]. However, these retinal abnormalities could not be confirmed during a subsequent examination by Hess [9]. Nonetheless, it has yet to be definitively established that the retina of amblyopes is absolutely normal.

The recent use of optical coherence tomography (OCT) has made it possible to quickly and non-invasively measure the retinal structure in humans. Spectral-domain OCT (SD-OCT) has especially improved the spatial resolution and scan speed, thereby enabling a detailed retinal analysis. Yen et al. [10] reported that the circumpapillary retinal nerve fiber layer (cpRNFL) of refractive amblyopic eyes was thicker than that observed in the normal fellow eyes when using time-domain OCT. The OCT findings reported by Li et al. [11] suggested that the foveal thickness in amblyopic eyes was greater than that in visually normal control eyes. However, it is still unclear as to why cpRNFL or foveal thickness in amblyopic eyes is thicker than that found in normal fellow eyes.

In addition, the use of the enhanced depth imaging (EDI) technique with SD-OCT has enabled imaging of the choroid [12]. The choroid accounts for 80-90% of the whole ocular blood flow and plays an important role in maintaining the retinal structure and function. Nishi et al. [13] used the EDI system with SD-OCT and reported that the subfoveal choroid of eyes with hyperopic anisometropic amblyopia was significantly thicker than that of the fellow eye and the age-matched controls. However, as of yet it has not been possible to use the EDI system with SD-OCT to measure the detailed choroidal thickness map using the 3D scan. In addition, another drawback of the EDI system is that it is difficult to clearly view the retina and choroid at the same time, as the retinal image quality decreases when we maximize the choroid image quality. Also, in order to be able to obtain a clear averaged image, it is necessary to acquire a large number of images. However, poor fixation during the acquisition time might preclude being able to perform successful scanning.

Recently, swept-source OCT (SS-OCT) has been used, as it can overcome the shortcomings of EDI. As SS-OCT is supposed to be able to reduce the poor images due to poor fixation, this means that a highly-detailed scan can be obtained even in the children with unstable fixation. Moreover, the use of the long central wavelength of 1,050 nm enables simultaneous visualization of the retina and the choroid. In addition, the use of the built-in automatic analysis software makes it possible to perform a map analysis of the choroidal thickness. However, to the best of our knowledge, there have been no reports that have used SS-OCT to investigate the retinal and choroidal thicknesses in amblyopia. Thus, the purpose of the current study was to use SS-OCT to evaluate the macular retinal and choroidal thickness in unilateral amblyopia due to anisometropia or strabismus.

## Methods

This study adhered to the tenets of the Declaration of Helsinki and was approved by the Institutional Review Board committee of Kawasaki Medical School. This study was designed as an observational case series and conducted from April 2013 until June 2016 in the Department of Ophthalmology at Kawasaki Medical School Hospital. Verbal informed consent for the examinations was obtained from each patient or one of the parents of each patient.

All of the enrolled patients were diagnosed with unilateral amblyopia and underwent SS-OCT examination. Ophthalmologic examinations performed in all patients included best-corrected visual acuity (BCVA), intraocular pressure, cycloplegic refraction, axial length (AL), cover and cover–uncover test, extraocular movements, slit-lamp, and funduscopy. The refraction was measured by the Auto Ref / Kerato / Tonometer RKT-7700 (NIDEK Co., Ltd., Gamagori, Japan). The AL was measured by an IOL Master device (Carl Zeiss Meditec AG, Jena, Germany).

Unilateral amblyopia was defined as a condition where the decimal BCVAs were less than 0.8 in the amblyopic eye due to anisometropia or strabismus and more than 1.0 in the fellow eye. For the statistical analysis, the decimal BCVA was transformed into a logarithm of the minimum angle of resolution (logMAR) unit. Anisometropia was defined as an interocular difference in refraction (spherical equivalent) of more than 2.0 diopters (D). Patients with strabismic amblyopia had manifest strabismus on the cover test and a spherical equivalent interocular difference in refraction of less than 2.0 D. The presence or absence of a history of amblyopia treatment was not considered. Patients with ocular disorders, a history of intraocular surgery, systemic disease that may have had an influence on the retinal or choroidal thickness, and children who did not sufficiently cooperate for the OCT examination were excluded from the study.

This study also enrolled 24 right eyes of 24 age-matched normal controls. The children in the control group had a decimal BCVA that was greater than 1.0, and did not have anisometropia, manifest strabismus, ocular disorders in either eye, or systemic disease that may have had an influence on the retinal or choroidal thickness.

### Measurement of the retinal and choroidal thickness

**Fig. 1:**
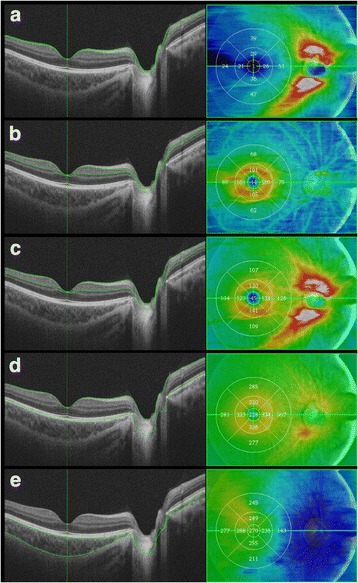
Retinal and choroidal thickness determined by swept-source optical coherence tomography. The retinal and choroidal thicknesses measured included (**a**) macular retinal nerve fiber layer, (**b**) ganglion cell layer + inner plexiform layer, (**c**) ganglion cell complex, (**d**) inner limiting membrane to the retinal pigment epithelium, and (**e**) choroidal thickness

SS-OCT was used to measure the retinal and choroidal thickness of the macula. The SS-OCT used for the measurements was the DRI OCT-1 Atlantis® (Topcon Corporation, Tokyo, Japan). The macular 3D scan (512 × 256 A scans/ 0.8 sec) program of the built-in automatic analysis software was used for the measurements of the retinal and choroidal thicknesses, which included the macular retinal nerve fiber layer (mRNFL), ganglion cell layer + inner plexiform layer (GCL+IPL), ganglion cell complex (GCC), inner limiting membrane to the outer border of the retinal pigment epithelium (ILM-RPE), and choroidal thickness (Fig. 1). The thickness from SS-OCT was corrected for magnification error using individual AL, spherical refraction, cylinder refraction, and corneal radius.

**Fig. 2:**
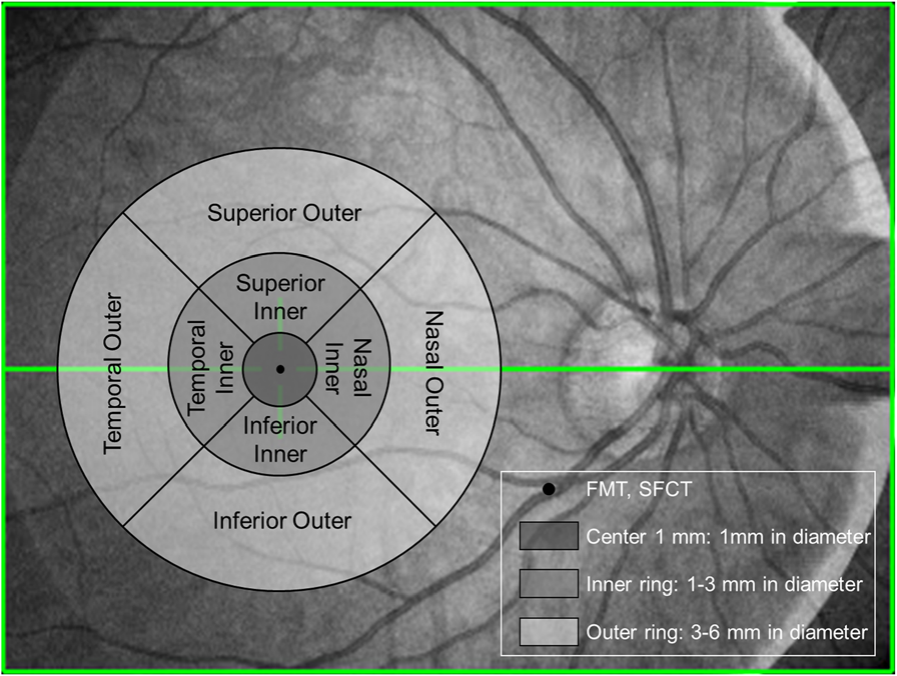
Analyzed regions of retinal and choroidal thickness using the Early Treatment Diabetic Retinopathy Study map. Three concentric macular regions were defined, with radii of 0.5 mm (center 1 mm), 0.5 to 1.5 mm (inner ring), and 1.5 to 3.0 mm (outer ring). Inner and outer rings were divided into four quadrants: superior, nasal, inferior, and temporal. Foveal minimum thickness and subfoveal choroidal thickness were also analyzed

The retinal and choroidal thickness was analyzed for each of the eyes in 9 regions of the macula in accordance with the Early Treatment Diabetic Retinopathy Study (ETDRS) [14]. Three concentric macular regions were defined, with radii of 0.5 mm (center 1 mm), 0.5 to 1.5 mm (inner ring), and 1.5 to 3.0 mm (outer ring). Inner and outer rings were divided into four quadrants: superior, nasal, inferior, and temporal. Foveal minimum thickness (FMT) and subfoveal choroidal thickness (SFCT) were also analyzed (Fig. 2).

An experienced technician (S.A.) performed all of the SS-OCT examinations after confirming the pupil diameter of the subjects was more than 4.0 mm. All SS-OCT examinations were performed between 9:00 AM and 12:00 PM to avoid any inclusion of diurnal variations in the choroidal thickness [15]. When there was a segmentation error in the automated analysis, S.A. performed a manual modification on the measurement of the choroidal thickness. However, the data in which segmentation was difficult to obtain due to signal attenuation were excluded from the study. The segmentation error was defined to be present if at least one of two experienced technicians (S.A. and K.G.) judged that the segmentation used for the measurements of the retinal or choroidal thickness was impossible.

### Statistical analyses

The statistical analysis was performed using the Bell Curve for Excel version 2.0 software program (Social Survey Research Information Co., Ltd., Tokyo, Japan). Data are presented as the means ± standard deviations. A one-way analysis of variance (ANOVA) was used to compare the average age, and a chi-square test was used to compare the genders among each of the groups (anisometropic amblyopia, strabismic amblyopia, and normal control groups). Multiple comparisons using the Bonferroni post hoc test were performed if there was a significant difference in the one-way ANOVA. The BCVA, refraction (spherical equivalent), and AL for the amblyopic, fellow and normal control eyes were compared using the paired and 2-sample t-tests. The average retinal or choroidal thicknesses among the amblyopic, fellow, and normal control eyes were compared by a one-way analysis of covariance (ANCOVA), which was controlled for the AL. The correlation of the differences for the retinal or choroidal thickness in the fovea or center 6 mm versus the differences in the BCVA between the amblyopic and fellow eyes was determined using Pearson's correlation coefficient. The correlation between the ILM-RPE thickness and the choroidal thickness in the fovea or center 6 mm was also determined using Pearson's correlation coefficient. The reproducibility of the judgment for the segmentation used in the measurements of the retinal or choroidal thickness was determined using kappa coefficient. For all of these analyses, *p*-values less than 0.05 were considered to be statistically significant.

## Results

### Demographic Data

This study enrolled 51 patients with unilateral amblyopia and 24 normal control subjects. All the subjects were Japanese. The study analyzed a total of 92 eyes of 46 patients with unilateral amblyopia and 24 right eyes of 24 normal control subjects (age: 7.8 ± 3.3 years). The study excluded 5 unilateral amblyopia patients due to the poor SS-OCT image quality. These five patients, in whom the segmentation of choroid from sclera was impossible, were associated with severe hyperopia. The reproducibility of the judgment for the segmentation error was excellent (κ = 0.88, *p* < 0.001). Among the 46 patients with unilateral amblyopia, there were 31 anisometropic amblyopes (age: 6.9 ± 3.8 years), and 15 strabismic amblyopes (age: 7.9 ± 4.2 years, with 11 patients exhibiting esotropia and 4 patients exhibiting exotropia).

Table 1 shows the clinical data for all of the subjects. No significant differences were observed between the anisometropic, strabismic, and normal control groups with regard to age (*p* = 0.54) and gender (*p* = 0.95).

**Table 1:** Demographic and clinical data for the amblyopia groups and the normal control group

	Anisometropic Group (*n* = 31)		Strabismic Group (*n* = 15)		Normal control Group (*n* = 24)
	AE	FE	AE	FE	NE
Age	6.9 ± 3.8 (3 to 18)		7.9 ± 4.2 (4 to 21)		7.8 ± 3.3 (3 to 16)
Gender (Male : Female)	13 : 18		6 : 9		9 : 15
Visual acuity (logMAR)	0.31 ± 0.22	-0.12 ± 0.08	0.16 ± 0.09	-0.10 ± 0.08	-0.12 ± 0.07
	(1.00 to 0.10)	(0.00 to -0.18)	(0.40 to 0.10)	(0.00 to -0.18)	(0.00 to -0.18)
Refraction (diopter)	5.02 ± 1.79	2.07 ± 1.79	3.28 ± 3.16	2.87 ± 3.10	0.44 ± 1.17
	(1.00 to 8.00)	(-1.00 to 5.25)	(-2.75 to 7.00)	(-3.25 to 6.75)	(-1.75 to 3.25)
Axial length (mm)	21.20 ± 0.95	22.21 ± 1.10	21.97 ± 1.17	21.99 ± 1.17	22.74 ± 1.06
	(19.61 to 23.89)	(20.73 to 24.91)	(20.30 to 24.42)	(20.40 to 24.08)	(20.85 to 24.61)

AE: Amblyopic eyes; FE: Fellow eyes; NE: Normal control eyes

Values are shown as mean ± standard deviation (range)

The logMAR in anisometropic or strabismic amblyopic eyes was significantly worse than that observed in the fellow eyes and normal control eyes (*p* < 0.001 for both comparisons). The logMAR in the anisometropic amblyopic eyes was significantly worse than that observed in the strabismic amblyopic eyes (*p* = 0.02).

The refraction in the anisometropic or strabismic amblyopic eyes was more hyperopic than that found for the corresponding fellow eyes (*p* < 0.001 for both comparisons), and was more hyperopic in the fellow eyes than in the normal control eyes (*p* < 0.001 for both comparisons).

In the anisometropic group, the AL in the amblyopic eyes (21.20 ± 0.95 mm) was shorter than that of the fellow eyes (22.21 ± 1.10 mm) (*p* < 0.001) and the normal control eyes (22.74 ± 1.06 mm) (*p* < 0.001), with no significant difference observed between the fellow and normal control eyes (*p* = 0.074). In the strabismic group, there was also no significant difference observed in the AL between the amblyopic (21.97 ± 1.17 mm) and the fellow eyes (21.99 ± 1.17 mm) (*p* = 0.80). In addition, the AL in the normal control eyes was larger than that found for the amblyopic (*p* = 0.038) and fellow eyes (*p* = 0.043).

### Macular inner retinal thickness

Tables 2, 3, 4, 5, 6 and 7 show the mean mRNFL, GCL+IPL, and GCC thicknesses in patients with unilateral amblyopia and the controls obtained when using SS-OCT.

**Table 2:** mRNFL thickness in the anisometropic amblyopia and normal control eyes

	Anisometropic amblyopia			*p* value^a^ (after adjusting for AL)		
	AE (*n* = 31)	FE (*n* = 31)	NE (*n* = 24)	AE vs FE	AE vs NE	FE vs NE
ETDRS maps						
Center 1 mm	2.2 ± 1.7	2.5 ± 1.8	2.8 ± 1.9	0.35	0.81	0.99
Inner ring (1-3 mm)						
Superior	26.4 ± 3.0	27.0 ± 3.3	28.7 ± 2.1	0.069	0.71	0.21
Nasal	20.6 ± 3.9	22.4 ± 2.8	24.3 ± 1.7	0.87	0.14	0.13
Inferior	26.6 ± 3.5	28.0 ± 4.0	29.4 ± 2.9	0.18	0.89	0.92
Temporal	20.2 ± 2.8	21.0 ± 2.6	22.1 ± 1.9	0.44	0.35	0.27
Outer ring (3-6 mm)						
Superior	40.6 ± 4.8	42.7 ± 4.8	42.1 ± 3.7	0.60	0.81	0.19
Nasal	46.6 ± 7.5	50.0 ± 7.7	50.9 ± 5.3	0.71	0.90	0.66
Inferior	41.6 ± 4.6	43.5 ± 5.6	43.3 ± 5.4	0.98	0.86	0.45
Temporal	24.2 ± 2.6	24.9 ± 2.6	25.8 ± 2.2	0.62	0.71	0.33
Center 6 mm	27.7 ± 2.9	29.1 ± 3.1	29.9 ± 1.9	0.59	0.50	0.96

**Table 3:** mRNFL thickness in the strabismic amblyopia and normal control eyes

	Strabismic amblyopia			*p* value^a^ (after adjusting for AL)		
	AE (*n* = 15)	FE (*n* = 15)	NE (*n* = 24)	AE vs FE	AE vs NE	FE vs NE
ETDRS maps						
Center 1 mm	3.5 ± 2.7	3.5 ± 3.5	2.8 ± 1.9	0.94	0.62	0.15
Inner ring (1-3 mm)						
Superior	27.9 ± 3.7	28.1 ± 2.8	28.7 ± 2.1	0.82	0.87	0.76
Nasal	24.2 ± 4.1	23.9 ± 3.1	24.3 ± 1.7	0.71	0.28	0.49
Inferior	28.7 ± 3.4	28.7 ± 4.1	29.4 ± 2.9	0.98	0.67	0.44
Temporal	21.6 ± 1.8	20.9 ± 3.2	22.1 ± 1.9	0.62	0.27	0.73
Outer ring (3-6 mm)						
Superior	40.1 ± 4.8	39.9 ± 2.5	42.1 ± 3.7	0.87	0.62	0.78
Nasal	53.1 ± 10.4	50.7 ± 9.6	50.9 ± 5.3	0.44	0.10	0.36
Inferior	42.8 ± 6.3	44.3 ± 8.6	43.3 ± 5.4	0.55	0.45	0.21
Temporal	24.9 ± 1.9	24.7 ± 2.8	25.8 ± 2.2	0.63	0.16	0.49
Center 6 mm	29.6 ± 3.7	29.4 ± 3.8	29.9 ± 1.9	0.82	0.49	0.62

AE: Amblyopic eyes; FE: Fellow eyes; NE: Normal control eyes; ETDRS: early treatment diabetic retinopathy study; AL: axial length

^a^ ANCOVA; ** *p* < 0.01; * *p* < 0.05

Values are shown as mean ± standard deviation (μm)

**Table 4:** GCL+IPL thickness in the anisometropic amblyopia and normal control eyes

	Anisometropic amblyopia			*p* value^a^ (after adjusting for AL)		
	AE (*n* = 31)	FE (*n* = 31)	NE (*n* = 24)	AE vs FE	AE vs NE	FE vs NE
ETDRS maps						
Center 1 mm	40.0 ± 7.0	41.8 ± 7.0	44.5 ± 6.3	0.77	0.41	0.38
Inner ring (1-3 mm)						
Superior	90.4 ± 5.3	90.8 ± 5.9	91.1 ± 4.7	0.069	0.053	0.68
Nasal	90.6 ± 5.6	91.6 ± 6.7	92.9 ± 4.9	0.11	0.059	0.93
Inferior	90.6 ± 5.7	91.2 ± 6.5	90.8 ± 5.1	0.10	0.23	0.29
Temporal	86.0 ± 7.1	87.1 ± 6.2	87.8 ± 5.2	0.062	0.24	0.55
Outer ring (3-6 mm)						
Superior	70.6 ± 6.3	67.4 ± 5.0	64.6 ± 5.9	0.11	0.21	0.35
Nasal	77.1 ± 7.2	73.0 ± 5.3	70.0 ± 6.6	0.24	0.18	0.21
Inferior	68.9 ± 8.5	64.1 ± 5.5	61.7 ± 7.1	0.26	0.26	0.45
Temporal	75.0 ± 6.5	72.7 ± 5.5	70.0 ± 5.7	0.43	0.41	0.87
Center 6 mm	76.6 ± 4.3	75.5 ± 3.9	74.8 ± 4.2	0.24	0.58	0.080

**Table 5:** GCL+IPL thickness in the strabismic amblyopia and normal control eyes

	Strabismic amblyopia			*p* value^a^ (after adjusting for AL)		
	AE (*n* = 15)	FE (*n* = 15)	NE (*n* = 24)	AE vs FE	AE vs NE	FE vs NE
ETDRS maps						
Center 1 mm	43.5 ± 9.3	45.1 ± 12.1	44.5 ± 6.3	0.65	0.63	0.27
Inner ring (1-3 mm)						
Superior	91.4 ± 5.3	91.6 ± 5.2	91.1 ± 4.7	0.88	0.93	0.79
Nasal	93.3 ± 7.0	92.7 ± 6.2	92.9 ± 4.9	0.96	0.29	0.23
Inferior	92.7 ± 5.3	90.9 ± 5.7	90.8 ± 5.1	0.32	0.18	0.29
Temporal	87.0 ± 5.6	87.5 ± 6.4	87.8 ± 5.2	0.83	0.78	0.49
Outer ring (3-6 mm)						
Superior	67.7 ± 6.4	69.0 ± 7.9	64.6 ± 5.9	0.97	0.98	0.98
Nasal	72.1 ± 8.1	75.5 ± 8.1	70.0 ± 6.6	0.19	0.88	0.15
Inferior	63.0 ± 6.5	65.9 ± 7.9	61.7 ± 7.1	0.17	0.80	0.43
Temporal	71.1 ± 5.4	73.0 ± 5.4	70.0 ± 5.7	0.83	0.30	0.21
Center 6 mm	75.8 ± 3.7	76.8 ± 3.7	74.8 ± 4.2	0.94	0.73	0.67

AE: Amblyopic eyes; FE: Fellow eyes; NE: Normal control eyes; ETDRS: early treatment diabetic retinopathy study; AL: axial length

^a^ ANCOVA; ** *p* < 0.01; * *p* < 0.05

Values are shown as mean ± standard deviation (μm)

**Table 6:** GCC thickness in the anisometropic amblyopia and normal control eyes

	Anisometropic amblyopia			*p* value^a^ (after adjusting for AL)		
	AE (*n* = 31)	FE (*n* = 31)	NE (*n* = 24)	AE vs FE	AE vs NE	FE vs NE
ETDRS maps						
Center 1 mm	42.4 ± 7.8	44.3 ± 7.5	47.3 ± 7.6	0.62	0.56	0.46
Inner ring (1-3 mm)						
Superior	116.7 ± 7.4	117.7 ± 8.1	119.8 ± 5.7	0.33	0.74	0.89
Nasal	111.8 ± 8.6	113.7 ± 8.8	117.1 ± 5.8	0.071	0.84	0.42
Inferior	117.2 ± 8.1	119.2 ± 9.8	120.2 ± 6.5	0.076	0.52	0.46
Temporal	105.7 ± 7.8	108.3 ± 7.6	110.0 ± 5.9	0.16	0.83	0.85
Outer ring (3-6 mm)						
Superior	110.8 ± 7.8	110.2 ± 7.6	106.6 ± 8.0	0.73	0.68	0.24
Nasal	123.9 ± 7.6	122.9 ± 7.9	121.0 ± 9.0	0.17	0.43	0.11
Inferior	110.5 ± 8.7	107.6 ± 7.3	104.9 ± 10.5	0.36	0.83	0.59
Temporal	99.2 ± 7.6	97.7 ± 6.6	95.8 ± 6.6	0.18	0.33	0.80
Center 6 mm	104.2 ± 5.8	104.6 ± 6.2	104.7 ± 5.3	0.11	0.38	0.48

**Table 7:** GCC thickness in the strabismic amblyopia and normal control eyes

	Strabismic amblyopia			*p* value^a^ (after adjusting for AL)		
	AE (*n* = 15)	FE (*n* = 15)	NE (*n* = 24)	AE vs FE	AE vs NE	FE vs NE
ETDRS maps						
Center 1 mm	47.2 ± 11.5	48.7 ± 14.8	47.3 ± 7.6	0.73	0.37	0.18
Inner ring (1-3 mm)						
Superior	119.3 ± 6.8	119.7 ± 6.8	119.8 ± 5.7	0.90	0.65	0.23
Nasal	118.2 ± 8.5	117.1 ± 8.6	117.1 ± 5.8	0.65	0.26	0.47
Inferior	121.3 ± 7.7	119.3 ± 8.7	120.2 ± 6.5	0.42	0.18	0.57
Temporal	108.7 ± 6.5	108.4 ± 7.4	110.0 ± 5.9	0.89	0.86	0.92
Outer ring (3-6 mm)						
Superior	107.7 ± 7.2	107.7 ± 5.5	106.6 ± 8.0	0.52	0.87	0.99
Nasal	125.1 ± 8.3	125.0 ± 7.3	121.0 ± 9.0	0.85	0.69	0.54
Inferior	105.7 ± 7.4	110.1 ± 8.2	104.9 ± 10.5	0.90	0.83	0.75
Temporal	96.3 ± 5.3	97.8 ± 5.6	95.8 ± 6.6	0.61	0.57	0.31
Center 6 mm	105.5 ± 5.3	106.0 ± 5.6	104.7 ± 5.3	0.81	0.43	0.36

AE: Amblyopic eyes; FE: Fellow eyes; NE: Normal control eyes; ETDRS: early treatment diabetic retinopathy study; AL: axial length

^a^ ANCOVA; ** *p* < 0.01; * *p* < 0.05

Values are shown as mean ± standard deviation (μm)

In both amblyopia groups, there was no significant difference in the mRNFL, GCL+IPL, and GCC thicknesses among the amblyopic, fellow, and normal control eyes for all of the sectors (Tables 2, 3, 4, 5, 6 and 7).

### Macular ILM-RPE thickness

Tables 8 and 9 shows the mean ILM-RPE thickness.

**Table 8:** ILM-RPE thickness in the anisometropic amblyopia and normal control eyes

	Anisometropic amblyopia			*p* value^a^ (after adjusting for AL)		
	AE (*n* = 31)	FE (*n* = 31)	NE (*n* = 24)	AE vs FE	AE vs NE	FE vs NE
FMT	181.6 ± 13.1	180.4 ± 13.0	186.1 ± 15.1	0.17	0.98	0.35
ETDRS maps						
Center 1 mm	218.9 ± 16.3	219.6 ± 14.8	226.2 ± 18.8	0.21	0.62	0.47
Inner ring (1-3 mm)						
Superior	310.6 ± 13.1	306.5 ± 12.3	309.0 ± 12.3	0.020*	0.094	0.073
Nasal	308.8 ± 14.9	305.9 ± 13.9	309.4 ± 14.1	0.047*	0.083	0.061
Inferior	307.1 ± 13.9	303.6 ± 13.6	304.7 ± 10.9	0.032*	0.46	0.82
Temporal	297.2 ± 14.3	295.0 ± 12.1	297.1 ± 12.3	0.059	0.21	0.15
Outer ring (3-6 mm)						
Superior	285.7 ± 13.4	278.2 ± 12.6	274.9 ± 14.8	0.065	0.59	0.22
Nasal	300.3 ± 13.2	291.2 ± 11.9	288.6 ± 16.1	0.32	0.35	0.10
Inferior	275.5 ± 14.7	265.6 ± 12.4	263.0 ± 15.5	0.13	0.45	0.85
Temporal	271.9 ± 13.3	263.2 ± 13.2	261.9 ± 12.2	0.16	0.32	0.73
Center 6 mm	286.6 ± 11.4	279.3 ± 10.4	278.1 ± 12.2	0.22	0.37	0.16

**Table 9:** ILM-RPE thickness in the strabismic amblyopia and normal control eyes

	Strabismic amblyopia			*p* value^a^ (after adjusting for AL)		
	AE (*n* = 15)	FE (*n* = 15)	NE (*n* = 24)	AE vs FE	AE vs NE	FE vs NE
FMT	187.7 ± 20.2	193.5 ± 24.6	186.1 ± 15.1	0.35	0.17	0.062
ETDRS maps						
Center 1 mm	228.5 ± 22.4	229.0 ± 25.3	226.2 ± 18.8	0.97	0.30	0.26
Inner ring (1-3 mm)						
Superior	313.1 ± 12.9	312.7 ± 13.7	309.0 ± 12.3	0.97	0.89	0.86
Nasal	315.1 ± 13.5	312.9 ± 13.2	309.4 ± 14.1	0.72	0.36	0.19
Inferior	311.0 ± 11.9	307.7 ± 11.9	304.7 ± 10.9	0.46	0.95	0.88
Temporal	300.0 ± 11.6	298.1 ± 12.9	297.1 ± 12.3	0.97	0.90	0.88
Outer ring (3-6 mm)						
Superior	281.4 ± 12.9	279.9 ± 13.3	274.9 ± 14.8	0.73	0.57	0.90
Nasal	298.7 ± 13.8	300.4 ± 13.0	288.6 ± 16.1	0.69	0.28	0.13
Inferior	267.8 ± 12.9	274.4 ± 14.6	263.0 ± 15.5	0.11	0.95	0.17
Temporal	266.3 ± 12.0	267.8 ± 12.9	261.9 ± 12.2	0.59	0.68	0.51
Center 6 mm	284.1 ± 10.8	285.3 ± 11.0	278.1 ± 12.2	0.73	0.45	0.30

AE: Amblyopic eyes; FE: Fellow eyes; NE: Normal control eyes; FMT: foveal minimum thickness; ETDRS: early treatment diabetic retinopathy study; AL: axial length

^a^ ANCOVA; ** *p* < 0.01; * *p* < 0.05

Values are shown as mean ± standard deviation (μm)

In the anisometropic group, the ILM-RPE thickness in the amblyopic eyes was thicker than that of the fellow eyes in the superior, nasal, and inferior sectors for the inner ring (*p* < 0.05 for all comparisons). However, there was no significant difference in the ILM-RPE thickness between the amblyopic and normal control eyes for all of the sectors. (Table 8)

In the strabismic group, there was no significant difference in the ILM-RPE thickness among the amblyopic, fellow, and normal control eyes for all of the sectors (Table 9).

### Macular choroidal thickness

Tables 10 and 11 shows the mean choroidal thickness.

**Table 10:** Choroidal thickness in the anisometropic amblyopia and normal control eyes

	Anisometropic amblyopia			*p* value^a^ (after adjusting for AL)		
	AE (*n* = 31)	FE (*n* = 31)	NE (*n* = 24)	AE vs FE	AE vs NE	FE vs NE
SFCT	320.8 ± 55.3	274.0 ± 55.2	274.7 ± 52.8	0.016*	0.017*	0.85
ETDRS maps						
Center 1 mm	320.2 ± 53.1	274.0 ± 53.3	277.4 ± 51.6	0.011*	0.044*	0.33
Inner ring (1-3 mm)						
Superior	306.2 ± 44.2	275.5 ± 52.7	279.6 ± 50.1	0.005**	0.080	0.31
Nasal	301.7 ± 53.5	244.7 ± 60.4	247.7 ± 51.5	0.013*	0.007**	0.33
Inferior	317.4 ± 51.4	265.3 ± 55.7	275.3 ± 45.3	0.005**	0.039*	0.18
Temporal	319.6 ± 51.4	286.8 ± 46.3	293.1 ± 54.8	0.037*	0.21	0.28
Outer ring (3-6 mm)						
Superior	293.6 ± 36.4	261.6 ± 45.0	265.0 ± 48.8	0.056	0.396	0.37
Nasal	251.1 ± 51.1	187.0 ± 59.9	185.7 ± 46.7	0.045*	0.003**	0.49
Inferior	287.1 ± 49.1	249.1 ± 46.3	260.4 ± 42.9	0.022*	0.41	0.16
Temporal	299.8 ± 49.3	278.7 ± 42.0	286.6 ± 54.2	0.21	0.84	0.37
Center 6 mm	290.2 ± 41.6	250.3 ± 44.5	255.7 ± 44.9	0.015*	0.049*	0.26

**Table 11:** Choroidal thickness in the strabismic amblyopia and normal control eyes

	Strabismic amblyopia			*p* value^a^ (after adjusting for AL)		
	AE (*n* = 15)	FE (*n* = 15)	NE (*n* = 24)	AE vs FE	AE vs NE	FE vs NE
SFCT	293.1 ± 74.3	274.1 ± 75.8	274.7 ± 52.8	0.78	0.94	0.78
ETDRS maps						
Center 1 mm	293.3 ± 68.1	279.0 ± 65.8	277.4 ± 51.6	0.99	0.92	0.90
Inner ring (1-3 mm)						
Superior	268.1 ± 40.3	287.6 ± 53.0	279.6 ± 50.1	0.22	0.70	0.73
Nasal	272.9 ± 60.8	265.0 ± 66.1	247.7 ± 51.5	0.73	0.76	0.85
Inferior	291.6 ± 67.5	280.6 ± 62.3	275.3 ± 45.3	0.72	0.87	0.91
Temporal	297.7 ± 57.2	292.3 ± 61.5	293.1 ± 54.8	0.98	0.96	0.93
Outer ring (3-6 mm)						
Superior	270.3 ± 39.8	278.6 ± 49.1	265.0 ± 48.8	0.53	0.49	0.92
Nasal	220.3 ± 56.5	215.3 ± 60.2	185.7 ± 46.7	0.80	0.22	0.41
Inferior	272.8 ± 55.9	267.0 ± 61.3	260.4 ± 42.9	0.79	0.94	0.77
Temporal	287.9 ± 51.8	291.7 ± 59.5	286.6 ± 54.2	0.86	0.86	0.72
Center 6 mm	268.1 ± 44.3	267.6 ± 50.8	255.7 ± 44.9	0.996	0.59	0.96

AE: Amblyopic eyes; FE: Fellow eyes; NE: Normal control eyes; SFCT: subfoveal choroidal thickness; ETDRS: early treatment diabetic retinopathy study; AL: axial length

^a^ ANCOVA; ** *p* < 0.01; * *p* < 0.05

Values are shown as mean ± standard deviation (μm)

In the anisometropic group, the choroidal thickness in the amblyopic eyes was significantly thicker than that of the fellow and normal control eyes in the SFCT, center 1 mm, nasal and inferior sectors of the inner ring, nasal sector of the outer ring, and center 6 mm (*p* < 0.05 for all comparisons), with no significant difference observed between the fellow and normal control eyes (Table 10).

In the strabismic group, there was no significant difference in the choroidal thickness among the amblyopic, fellow, and normal control eyes for all of the sectors (Table 11).

### Correlation between the difference of the BCVA and the difference of the retinal or choroidal thickness in the amblyopic and fellow eyes

In the anisometropic group, the difference in the logMAR was not significantly correlated with the difference in the mRNFL thickness (*r* = 0.25, *p* = 0.18), GCL+IPL thickness (*r* = -0.12, *p* = 0.52), GCC thickness (*r* = 0.037, *p* = 0.84), ILM-RPE thickness (*r* = -0.13, *p* = 0.47), FMT (*r* = -0.31, *p* = 0.094), and SFCT (*r* = 0.27, *p* = 0.15). Only the difference in the choroidal thickness was significantly correlated with the difference in the logMAR (*r* = 0.37, *p* = 0.039). In addition, there was no significant correlation between the FMT and SFCT (amblyopic eyes: *r* = -0.33, *p* = 0.069, fellow eyes: *r* = -0.19, *p* = 0.31) or the ILM-RPE and choroidal thickness in the center 6 mm (amblyopic eyes: *r* = -0.23, *p* = 0.21, fellow eyes: *r* = -0.085, *p* = 0.65).

In the strabismic group, the difference in the logMAR was not significantly correlated with the difference in the mRNFL thickness (*r* = 0.20, *p* = 0.47), GCL+IPL thickness (*r* = -0.058, *p* = 0.84), GCC thickness (*r* = 0.46, *p* = 0.085), ILM-RPE thickness (*r* = 0.20, *p* = 0.47), choroidal thickness (*r* = 0.048, *p* = 0.86), FMT (*r* = -0.076, *p* = 0.79), and SFCT (*r* = -0.53, *p* = 0.36). In addition, there was no significant correlation between the FMT and SFCT (amblyopic eyes: *r* = -0.21, *p* = 0.44, fellow eyes: *r* = -0.33, *p* = 0.22) or the ILM-RPE and choroidal thickness in the center 6 mm (amblyopic eyes: *r* = 0.22, *p* = 0.43, fellow eyes: *r* = 0.24, *p* = 0.39).

## Discussion

This study using SS-OCT measurements demonstrated that inner retinal thickness was not found to be significantly altered in unilateral amblyopia and that the choroidal thickness of amblyopic eyes in unilateral amblyopia exhibited different distinctive characteristics depending on the type of amblyopia. Specifically, in anisometropic amblyopia, the choroidal thickness in the amblyopic eyes was significantly thicker than that of the fellow and normal control eyes. In contrast, in strabismic amblyopia, there was no significant difference in the retinal or choroidal thicknesses among the amblyopic, fellow, and normal control eyes.

A large number of studies have recently used SD-OCT to assess the thickness of the retina [11] or choroid [13, 16–22] in unilateral amblyopia. However, none of the previous reports have used SS-OCT to analyze the retinal and choroidal thickness at the same time. Furthermore, these previous studies manually performed the choroidal thickness measurements only at specific points and not over defined areas [13, 16–22]. Thus, the current study is the first to use SS-OCT to evaluate the averaged regional retinal and choroidal thickness in unilateral amblyopia using a 3D map.

In addition, previous studies that used OCT to examine the retinal or choroidal thicknesses of normal eyes have reported that the retinal thickness was influenced by age or sex, while the choroidal thickness was influenced by age, sex, AL, or refraction [23–26]. However, our current study found no significant difference in terms of sex or age between the anisometropic amblyopia, strabismic amblyopia, and normal control groups. Although the AL was significantly shorter in the anisometropic amblyopic eyes versus the fellow or normal control eyes, we compared the retinal or choroidal thickness using a statistical technique that was controlled for the AL.

### Macular inner retinal thickness

Previous studies that used SD-OCT to evaluate the inner retinal thickness reported finding no significant differences in the GCL+IPL or GCC thicknesses between the amblyopic eyes and the fellow and normal control eyes [16, 27–29]. In contrast, Park et al. [30] reported that the GCL+IPL thickness was thinner in amblyopic eyes versus the fellow eyes. Tugcu et al. [31] further reported that the GCC thickness in anisometropic amblyopia was thicker in amblyopic eyes versus controls, while in strabismic amblyopia, it was thinner in the amblyopic eyes versus the controls. In the current study, there were no significant differences in the mRNFL, GCL+IPL, and GCC thickness found among the amblyopic, fellow, and normal control eyes.

Yen et al. [10] reported that while the RNFL thickness might be affected by refractive amblyopia, it was not affected by strabismic amblyopia. Yen et al. [10] hypothesized that amblyopia might affect the postnatal maturation of the retina, including the postnatal reduction of the optic nerve axons [32, 33], which could then lead to a measurable increase in the RNFL thickness in amblyopic eyes. However, it has also been reported that the decrease of the optic nerve axons in humans stabilizes around week 29 of gestation [32]. In addition, Potts et al. [33] reported that the number of retinal ganglion cells in newborn rats decreased to the same number as the number of retinal ganglion cells in adult rats on the 10th postnatal day. Therefore, neither anisometropic nor strabismic amblyopia, which exhibit a much later onset after birth, will likely prevent this normal development.

### Macular ILM-RPE thickness

There have been many previous studies that have examined the macular ILM-RPE thickness of unilateral amblyopia [11, 27–31, 34–39]. Li et al. [11] performed a meta-analysis and reported that the FMT and the thickness of the center 1 mm and 6 mm in the amblyopic eyes were significantly greater than that observed in the fellow eyes (4.6 μm, 3.2 μm, and 3.5 μm, respectively). Additionally, only the FMT was significantly increased in the amblyopic eyes as compared with the normal control eyes.

In the anisometropic group, our current study showed that the ILM-RPE thickness in the amblyopic eyes was thicker than that of the fellow eyes only in the superior, nasal, and inferior sectors for the inner ring. However, there was no significant difference in the ILM-RPE thickness between the amblyopic and normal control eyes for all of the sectors. In addition, there was no significant difference between the fellow and normal control eyes. Thus, there is no clear explanation for our results of the ILM-RPE thickness in anisometropic amblyopia. In the strabismic group, there was also no significant difference in the ILM-RPE thickness among the amblyopic, fellow, and normal control eyes in all of the sectors.

Al-Haddad et al. [34] found that the central macular thickness was significantly increased in amblyopic eyes as compared to that of the fellow eyes in anisometropic amblyopia, but not in strabismic amblyopia. Park et al. [30] additionally reported finding no statistically significant differences in the total macular thickness between the amblyopic and normal fellow eyes in unilateral amblyopia, with these eyes also showing no significant differences in the refractive errors. Based on these findings, we believe that it is possible that a change in the ILM-RPE thickness of amblyopic eyes relates to refraction. However, Al-Haddad et al. [34] also reported that anisometropia alone did not lead to such a difference, which suggests that there could possibly be a correlation between amblyopia and the development of the retinal layers.

With regard to the FMT, Huynh et al. [35] reported that amblyopic eyes had a slightly greater foveal minimum thickness than the fellow and normal control eyes in unilateral amblyopia, which was due to the anisometropia and strabismus. In addition, they found that the inner macular ring was thinner in the amblyopic versus the normal fellow eyes. As a result, these authors proposed a hypothesis that the arrest of normal postnatal changes would most likely affect the normal maturation of the macula, including the movement of Henle’s fibers away from the foveola, along with a decrease in the foveal cone diameter. However, there are many other reports that have found that the FMT of amblyopic eyes was no different from the fellow and normal control eyes [20, 37, 38]. The findings of our current study do not support Huynh et al.’s hypothesis [35], as we found that there were no changes in the FMT of amblyopic eyes.

Huynh et al. [35] additionally reported finding that the foveal thickening in the amblyopic eyes was more remarkable in the no treatment versus the treatment group. Pang et al. [36] also examined the FMT of amblyopic eyes and reported that it became thinner after treatment as compared to before treatment, although there was no correlation found between the FMT and VA improvement. The disagreement between our current results and the previous studies [11, 35, 36] might be due to the fact that our present study did not exclude patients who had a history of amblyopia treatment. Additional studies that investigate classified amblyopes according to refractive error, past history of amblyopia treatment, and the depth of amblyopia will need to be undertaken in order to clarify these effects in the future.

Using the ETDRS map, Wu et al. [39] found that the macular thickness in the center 1 mm, inner ring, or outer ring was not significantly different between the amblyopic and fellow eyes in hyperopic anisometropic amblyopia. Kim et al. [28] additionally reported that while the macular thickness (inner temporal, outer superior, outer nasal, and outer inferior) in amblyopic eyes was significantly greater than that found in the fellow eyes, these thicknesses were not significantly different between the amblyopic and normal control eyes. Our current study also found that there were no significant differences in the ILM-RPE thickness between the amblyopic and normal control eyes for all of the sectors, similar to that reported in previous studies [28, 39].

In addition, we found that there were no significant differences in the ILM-RPE thickness between the amblyopic and normal control eyes, and between the fellow and normal control eyes, although our study did reveal that the ILM-RPE thickness exhibited a significant difference between the amblyopic and fellow eyes in only a few regions. Thus, while the difference of the ILM-RPE thickness in a few regions of the inner ring between the amblyopic and fellow eyes was statistically significant, the clinical significance appears to be trivial.

### Macular choroidal thickness

In a preliminary study, Nishi et al. [13] reported that the subfoveal choroid of eyes with hyperopic anisometropic amblyopia was significantly thicker than that of the fellow eye and the age-matched controls. Many other investigators have reported that the SFCT in amblyopic eyes was greater than that of the fellow eyes with hypermetropic anisometropic amblyopia [17–22]. However, there are only a few studies that have examined the choroidal thickness in accordance with the amblyopic cause [17, 18, 22]. Similar to that for retinal thickness, whether "amblyopia influences" [13, 17] or "refraction influences" [40] are associated with the choroid thickening in amblyopic eyes remains controversial.

In the anisometropic group, our current study showed that the choroidal thickness in the amblyopic eyes was significantly thicker than that of the fellow and normal control eyes in the SFCT, center 1 mm, nasal and inferior sectors of the inner ring, nasal sector of the outer ring, and the center 6 mm. There was no significant difference between the fellow and normal control eyes. In the strabismic group, there was also no significant difference in the choroidal thickness among the amblyopic, fellow, and normal control eyes for any of the sectors.

Nishi et al. [13] described the possibility that the increased SFCT of amblyopic eyes is under the influence of the amblyopia, as the profile of the choroidal thickness in the amblyopic eyes was different from that of the fellow eyes and control eyes. In amblyopic eyes, the choroid was the thickest in the subfoveal area followed by the temporal sector, with the thinnest area found in the nasal sector. The authors hypothesized that the ocular compensation and choroidal accommodation [41] for the hyperopic defocus was suppressed in amblyopic eyes, which resulted in an increased SFCT. Since all the anisometropic amblyopia eyes in our current study had hyperopic defocus, our results do not contradict their hypothesis [13].

On the other hand, Xu et al. [17] suggested the possibility that FMT thickening is associated with the SFCT thickening of amblyopic eyes. Thus, a thicker retina would likely require a greater blood supply. If so, then the choroid might thicken in order to be able to supply more blood to the outer retina. However, the article by Xu et al. [17] did not report the retinal thicknesses found in their investigation. Although the FMT of the anisometropic amblyopia group did not exhibit a significant difference between the amblyopic and fellow eyes, the SFCT was thicker in the amblyopic eyes in our study. Moreover, we investigated the correlation between the FMT and SFCT, or ILM-RPE and choroidal thickness in the center 6 mm in the anisometropic amblyopia group, but found no significant correlations. Therefore, we do not believe that the thickening of the choroid occurs directly following the retinal thickening.

In the strabismic amblyopia group, our study showed that there was no significant difference in the choroidal thickness among the amblyopic, fellow, and normal control eyes. This was a novel finding as compared to previous studies that have reported that the choroid of amblyopic eyes was thick in strabismic amblyopia [17, 18, 22]. With regard to this discrepancy between the previous reports and our current study, it is possible that refraction differences between the amblyopic and fellow eyes could have influenced the results. However, similar to the strabismic amblyopia group in the current study, the findings of the previous reports [17, 18, 22] were not complicated by the presence of anisometropia. Alternatively, there is a possibility that differences in the measurement procedures used to determine the choroidal thickness (analysis of the distance between two points set manually versus mapping analysis) could have had an effect on the choroidal thickness obtained. To the best of our knowledge, there have been no previous studies that have used SS-OCT to map the choroidal thickness. The choroidal thickness analysis in the previous studies used data that were measured as the distance from one point to another. However, these types of measurements could be easily affected by minute changes of the point actually measured. In contrast, as our present study used a map analysis, this should have resulted in more precise measurements, thereby generating more reliable data for the choroidal thickness.

In this study, changes of the choroidal thickness in amblyopic eyes were the most remarkable in the nasal region. This result was similar to the past reports [13, 17, 22]. However, it remains unclear as to why the only place that the choroidal thickening was not seen was in the temporal region in anisometropic amblyopia.

### Correlation between difference of the BCVA and the difference of the retinal or choroidal thickness in the amblyopic and fellow eyes

Various studies have investigated the correlation of the retinal thickness and VA in eyes with amblyopia and reported that the VA was not correlated with the retinal thickness regardless of amblyopia type [29, 36, 42]. Our current results, which demonstrated that retinal thicknesses measured by OCT were unrelated to the degree of amblyopia, appear to support the findings of previously published reports in the literature [29, 36, 42].

Pang et al. [36], who reported finding that there was no correlation between FMT and VA improvement, speculated that the reason for these results was that the critically important factor that is required in high-level acuity is the foveal cone density and not the foveal thickness. In contrast, Nishi et al. [38] reported that optical treatments resulted in an improvement of the VA and also a lengthening of the outer segment in anisohypermetropic amblyopic eyes. In addition, they also found a significant correlation between the increased outer segment length and better BCVA. Based on these findings, it is possible that there are minute structural changes that occur and cannot be detected by simply measuring the retinal and choroidal thicknesses that occur in amblyopic eyes.

On the other hand, a few studies reported no correlation between the VA and choroidal thickness [17, 40]. However, in our current study, as there was a weak correlation between the difference for the choroidal thickness and the difference for the BCVA between the amblyopic and fellow eyes, the choroidal thickening in anisometropic amblyopia in the center 6 mm found may be related to the change in the visual function that is observed in amblyopia. A further study is necessary whether our results were due to visual function or secondary to refraction differences between the eyes.

### Limitation

As a limitation for the current study, we were not able to match the refraction of the normal control eyes to that of the amblyopic eyes. If this had been possible, we might have been able to clearly determine whether the retinal or choroidal thickening was caused by hyperopia or by the amblyopia itself. However, finding a group of such controls is difficult, as eyes with high hyperopia should produce amblyopia to some extent during the time that they are uncorrected for the refractive error after birth.

## Conclusion

We found no significant difference in inner retinal thickness of the patients with unilateral amblyopia. Although there were significant interocular differences for the macular choroidal thicknesses in the hyperopic anisometropic amblyopia patients, there was no significant interocular difference observed in strabismic amblyopia. The results of this study do not support the hypothesis that the change was simply due to the differences in refractive error, as we used a statistical analysis that takes the AL into consideration. Thus, the noted differences may be due to a combination of the difference in the refraction and that of the pathogenesis of the disease between the two types of amblyopia.

## Abbreviations

AE: Amblyopic eyes
AL: Axial length
ANOVA: A one-way analysis of variance
BCVA: Best-corrected visual acuity
cpRNFL: Circumpapillary retinal nerve fiber layer
EDI: Enhanced depth imaging
ETDRS: Early treatment diabetic retinopathy study
FE: Fellow eyes
FMT: Foveal minimum thickness
GCC: Ganglion cell complex
GCL+IPL: Ganglion cell layer + inner plexiform layer
ILM-RPE: Inner limiting membrane to the retinal pigment epithelium
log MAR: Logarithm of the minimum angle of resolution
mRNFL: Macular retinal nerve fiber layer
NE: Normal control eyes
SD-OCT: Spectral-domain optical coherence tomography
SFCT: Subfoveal choroidal thickness
SS-OCT: Swept-source optical coherence tomography
